# Medium and long-term radiographic and clinical outcomes of Dynesys dynamic stabilization versus instrumented fusion for degenerative lumbar spine diseases

**DOI:** 10.1186/s12893-023-01943-6

**Published:** 2023-02-28

**Authors:** Lu-Ping Zhou, Ren-Jie Zhang, Jia-Qi Wang, Hua-Qing Zhang, Jin Shang, Yang Gao, Chong-Yu Jia, Jing-Yu Ding, Lai Zhang, Cai-Liang Shen

**Affiliations:** 1 Department of Orthopedics and Spine Surgery, The First Affiliated Hospital of Anhui Medical University, 218 Jixi Road, Hefei, 230022 Anhui China; 2 Department of Radiology, The First Affiliated Hospital of University of Science and Technology of China, 107 Huanhudong Road, Hefei, 230031 Anhui China; 3 Outpatient Department, The 55th Retired Cadres of the Beijing Garrison of PLA, 4 Wanshou Road, Beijing, 100036 China

**Keywords:** Dynamic stabilization, Dynesys, Fusion, Lumbar, Pedicle screw

## Abstract

**Background:**

Dynesys stabilization (DS) is utilized to preserve mobility at the instrumental segments and prevent adjacent segment pathology in clinical practice. However, the advantages of DS method in medium and long-term follow-up remain controversial.

**Objective:**

To compare the radiographic and clinical outcomes between DS and instrumented fusion in the treatment of degenerative lumbar spine disease with or without grade I spondylolisthesis with a minimum follow-up period of 2 years.

**Methods:**

We conducted a comprehensive search of PubMed, EMBASE, Cochrane, and Web of Science databases, Chinese National Knowledge Databases, and Wanfang Database for potentially eligible articles. Clinical outcomes were assessed in terms of VAS and ODI scores, screw loosening and breakage, and surgical revision. Radiographic outcomes were assessed in terms of postoperative range of movement (ROM) and disc heigh. Moreover, adjacent segment degeneration (ASDeg) and adjacent segment disease (ASDis) were evaluated.

**Results:**

Seventeen studies with 1296 patients were included in the meta-analysis. The DS group was associated with significantly lower postoperative VAS scores for low-back and leg pain, and lower rate of surgical revision than the fusion group. Moreover, the Dynesys group showed significantly less ASDeg than the fusion group but showed no significant advantage over the fusion group in terms of preventing ASDis. Additionally, the ROM at the stabilized segments of the fusion group decreased significantly and that at the adjacent segments increased significantly compared with those of the DS group.

**Conclusion:**

DS showed comparable clinical outcomes and provided benefits in preserving the motion at the stabilized segments, thus limiting the hypermobility at the adjacent segments and preventing ASDeg compared with the fusion method in degenerative disease with or without grade I spondylolisthesis.

**Supplementary Information:**

The online version contains supplementary material available at 10.1186/s12893-023-01943-6.

## Introduction

Lumbar fusion has been considered as the gold standard for the treatment of lumbar degenerative pathologies over the last decades [1, 2]. However, instrumented fusion causes a series of complications, including instrumentation failure, pseudoarthrosis, and pain in donors [3–5]. Moreover, the preservation of lumbar motion is less considered in fusion instrument, thus limiting the motility of stabilized segments and the increased load on adjacent segments, which may increase the risk of adjacent segment degeneration (ASDeg), adjacent segment disease (ASDis), and severe post-operative functional disabilities [6–8].

In recent years, non-fusion systems have been applied clinically to prevent the incidence of adjacent segment pathology (ASP, including ASDeg and ASDis) after lumbar surgeries [9–11]. The Dynesys posterior dynamic stabilization system (Zimmer Inc., Warsaw, IN, USA), which was first introduced in 1994, is a commonly used dynamic stabilization device [12]. This system consists of pedicle screws (Ti alloy), polyethylene–terephtalate (PET) cords, and polycarbonate–urethane (PCU) spacers for the stabilization of stabilized segments, restoration of normal segmental kinematics, and preservation of adjacent motion, and these parts aim to prevent the instability and decrease ASP incidence [5, 13, 14]. The Dynesys system (DS) has shown significant improvement in terms of visual analog scale (VAS) pain scores, Oswestry disability index (ODI) scores, trauma severity, and recovery time compared with the fusion method [4, 15–18]. Moreover, the range of movement (ROM) at adjacent segments and along with the load across the intervertebral and adjacent discs has been reduced [4, 7, 13, 15, 19].

However, inconsistent results have been obtained in the advantages of DS over fusion method in terms of clinical outcomes, including the relief in post-operative pain status (VAS scores) and functional status restoration (ODI scores) [3, 5–7, 20–23]. Whether DS can retain ROM at surgical segment and reduce ASP in long-term follow-up remains controversial [5, 6, 23]. Furthermore, previous studies have mainly focused on short-term clinical efficiency, and limited reports have focused on medium and long-term outcomes with conflicting findings, especially trials involving fusion method as control [7, 15, 24–26]. Thus, this meta-analysis aimed to compare the radiographic and clinical outcomes of dynamic DS and instrumented fusion for the treatment of degenerative lumbar spine diseases with a minimum follow-up period of 2 years.

## Methods

### Search strategy

This meta-analysis was performed according to the Preferred Reporting Items for Systematic Reviews and Meta-analyses guidelines [27]. A systematic search on PubMed, EMBASE, Cochrane, Web of Science databases, Chinese National Knowledge Databases, and Wanfang Database was conducted to identify potentially eligible articles from inception up until November 14, 2022 without language restriction. The terms “Dynesys” or “dynamic” or “semi-rigid” and “fusion” and “lumbar” were used for the search. The detailed search strategy is summarized in Additional file 1. Two reviewers independently searched all the titles and abstracts. The reference lists of relevant studies on DS were also reviewed for additional literature. Full-text articles were obtained when uncertainties were encountered. Any disagreement was settled by a third reviewer.

### Inclusion and exclusion criteria

The inclusion and exclusion of studies for the meta-analysis were based on the following criteria. (1) For participants, the study population consisted of patients who satisfied the following criteria: aged 18 years or older; mean follow-up period ≥ 2 years; suffering from lumbar degenerative diseases, including disc herniation, lumbar spinal stenosis, and grade I degenerative spondylolisthesis; and having 1–4 fixed segments in the lumbar. Studies on patients with grade II or higher spondylolisthesis, ankylosis spondylitis, spinal tumor, and severe spinal deformity were excluded. (2) The intervention in the experimental group was dynamic DS. Studies on hybrid dynamic stabilization and other kinds of dynamic stabilization, including Coflex, Wallis, and X-stop systems were excluded. (3) For comparison, the intervention in the control group was instrumented fusion methods, including posterior lumbar interbody fusion, transforaminal lumbar interbody fusion (TLIF), and posterolateral fusion. (4) In terms of outcomes, studies were eligible if they satisfied at least one of the following outcomes: clinical outcomes at final follow-up (VAS and ODI scores, screw loosening and breakage, surgical revision), ASP (ASDeg and ASDis), and radiographical outcomes (postoperative ROM and disc heigh). ASDeg (radiographic ASD) represents radiographic etiologies adjacent to the surgically treated spinal level that involves loss of disc height, disc degeneration, stenosis, instability, or hypertrophic facet arthritis, regardless of the presence of symptoms. [8] ASDis (symptomatic ASD) is a clinical symptom (manifested as pain, numbness, or the other symptoms caused by nerve compression) that is correlated with radiographic changes in adjacent segments. [2, 8] The primary outcomes considered were radiographic outcomes and ASP. (5) For study design, randomized controlled trials (RCTs) or comparative studies were eligible. Case series, case reports, reviews, and conference reports were excluded.

### Risk of bias assessment

Cochrane risk-of-bias criteria [28] and Newcastle–Ottawa Scale (NOS) [29] were used to assess the methodological quality of the included RCTs and comparative studies. The included randomized controlled trials were evaluated based on randomization sequence generation, allocation concealment, blinding of participants and personnel, blinding of outcome assessment, incomplete outcome data, selective reporting, and other bias. We defined other biases as different baseline characteristics in the experimental and control groups. The bias of domains was qualified as low risk, high risk, or unclear risk. Meanwhile, comparative cohort studies were assessed in terms of selection of patients, comparability, and outcomes of the case and control groups, with scores ranging from 0 to 9. The studies were evaluated as high quality (score 8 or 9), moderate quality (score 6 or 7), and low quality (score 5 or less).

### Data extraction

Two reviewers independently extracted relevant data from the qualified studies. Any disagreement was settled by a third reviewer. Leading author, publication year, study design, country of origin, study period, age, gender distribution, number of treated segments, fusion type, and follow-up period were extracted from the included studies.

### Statistical analysis

Odds ratio (OR) and its 95% confidence interval (CI) were calculated for dichotomous data. Mean difference (MD) and its 95% CI were calculated for continuous data. We used I^2^ and chi-squared tests at a significance level of *P* < 0.05 for assessment of statistical heterogeneity. A fixed-effects model was utilized if no evidence of heterogeneity (I^2^ < 50%) was observed among the studies. Otherwise, a random-effects model was used. The sensitivity analyses were performed to investigate the source of heterogeneity. In addition, publication bias was evaluated by Egger tests when the number of included studies was 9 or more. Except for publication bias assessed by STATA version 15.1, statistical analysis was performed with Review Manager version 5.3. All tests were two-tailed, and *P* < 0.05 was considered statistically significant.

## Results

### Study search

**Fig. 1:**
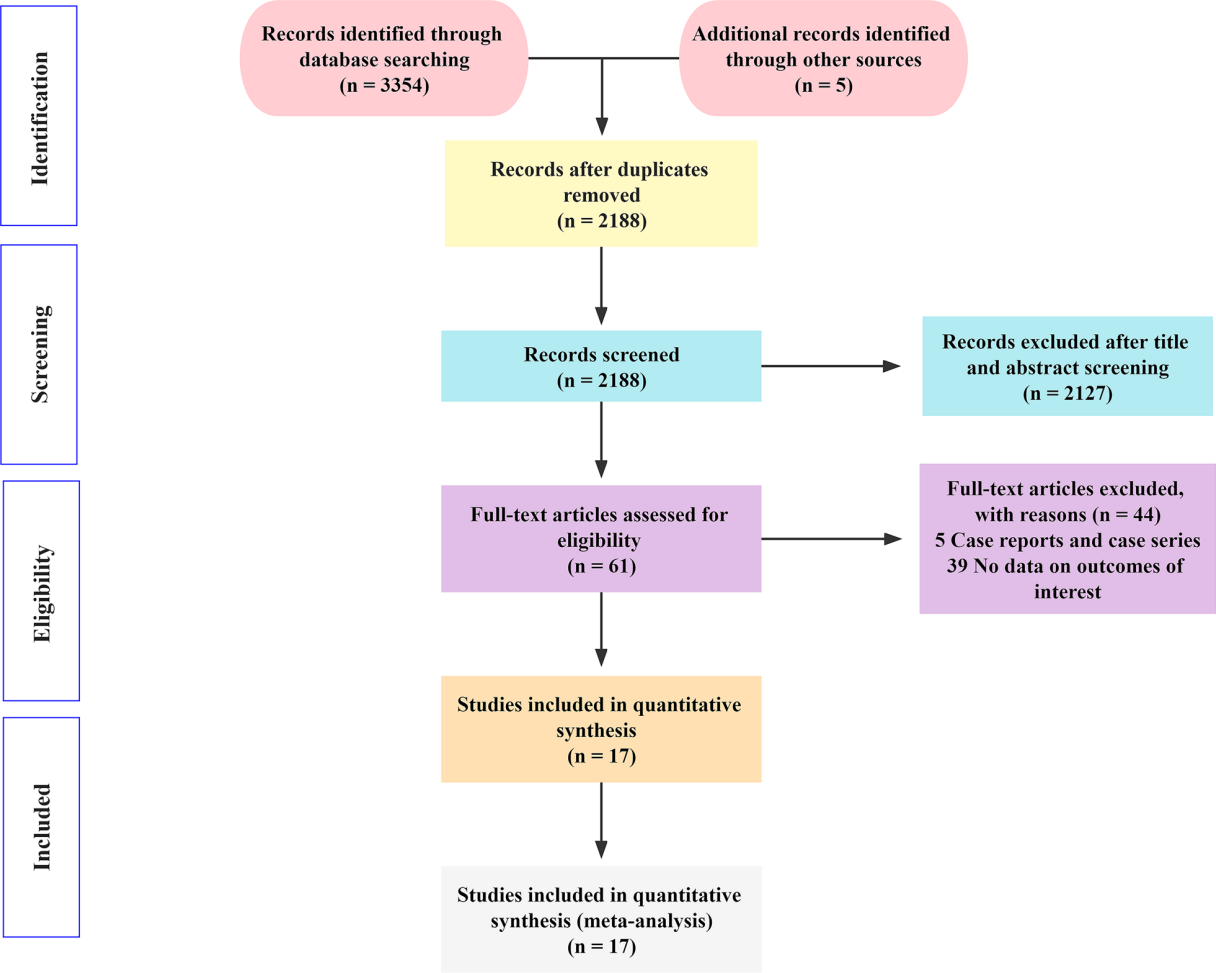
Flow diagram of the study selection

The process for literature search is summarized in Fig. 1. A total of 3359 potential papers were inspected from the electronic searches, and 1171 studies were excluded because of duplication. After assessing the titles and abstracts, 2127 studies were removed, and the 61 remaining articles were downloaded for full-text verification. Finally, one prospective randomized controlled trial [21], five prospective clinical studies [3, 15–17, 30], and 11 retrospective studies [4, 7, 22, 24–26, 31–35] were deemed eligible and included in the meta-analysis.

### Main characteristics of the included studies

Table 1 summarizes the main characteristics of the included studies. The baseline information of the 17 studies was balanced and comparable. Among the included studies, 14 studies [3, 4, 7, 15–17, 21, 22, 24, 30–34] were conducted in China, and one study was conducted in France [35], Italy [25], and the UK [26].

**Table 1:** Characteristics of included studies

Study	Study design	Country	Study Period	Age (years)		Sex (M/F)		No. of treated segments	Type of fusion	Follow-up(months)	
				Dynesys	Fusion	Dynesys	Fusion			Dynesys	Fusion
Luo et al., 2022	Retro	China, Chongqing	2013–2017	62.8 ± 11.6	58.2 ± 12.2	11/23	9/22	NA	TLIF	44 (36—78)	39 (30—73)
Zheng et al., 2021	Retro	China, Beijing	2012–2017	67.67 ± 5.70	66.98 ± 6.11	16/24	15/24	2–3	PLIF	41.90 ± 9.89	43.68 ± 13.82
Zhang et al., 2021	Retro	China, Zhejiang	2010–2012	55.1 ± 14.6	52.6 ± 16.8	14/9	12/8	2	PLIF	78.0 ± 25.6 (60–120)	
Hu et al., 2019	Retro	China, Shanghai	2011–2013	69.27 ± 4.92	69.41 ± 4.23	11/11	22/22	1–3	TLIF	68.50 ± 6.40	70.14 ± 7.26
Ren et al., 2018	Retro	China, Sichuan	2011–2015	49.5 ± 11.6	52.7 ± 12.4	27/11	31/13	1	TLIF	25.4 ± 4.0	24.8 ± 5.2
Kuo et al., 2018	Retro	China, Taiwan	2007–2014	60.1 ± 10.8	58.0 ± 11.7	21/35	8/15	1	TLIF	35.2 (24–89)	
Wu et al., 2017	Pro	China, Zhejiang	2008–2010	49.6 ± 8.3	52.5 ± 6.9	14/12	18/22	2–4	PLIF	50.3 (46—65)	52.8 (48 – 68)
Liu et al., 2017	Pro	China, Beijing	2011–2013	52.9 ± 10.3	54.6 ± 9.9	26/29	29/29	1	PLIF + TLIF	24	
Bredin et al., 2017	Retro	France, Marne	2005–2011	60.3 ± 9.4	61.3 ± 8.4	18/15	13/12	1–2	PLF	93.6 ± 16.5	91.7 ± 13.5
Zhang et al., 2016	Retro	China, Beijing	2008–2011	48.1 ± 12.3	52.3 ± 12.9	31/15	37/13	1–3	PLIF	53.6 ± 5.3	55.2 ± 6.8
He et al., 2016	Pro	China, Jiangsu	NA	58.3 ± 13.5	61.4 ± 15.2	7/5	9/15	1–2	PLIF	28.7 ± 5.3	30.1 ± 6.8
Wang et al., 2016	Retro	China, Liaoning	2009–2013	42.8 ± 6.7	56.8 ± 6.2	24/21	17/23	2–3	PLIF	30.6 ± 8.6	32.7 ± 7.9
Fei et al., 2015	Pro	China, Jiangsu	2007–2009	47.3 ± 12.9	52.9 ± 11.2	51/44	40/41	1–3	PLIF	36–66	
Yang et al., 2014	Pro	China, Shanghai	2010–2012	55.96 ± 7.68	54.69 ± 3.26	17/13	21/24	1–2	PLIF	26.64 ± 5.16	26.04 ± 9.12
Silvestre et al., 2014	Retro	Italy, Bologna	2002–2005	68.4 (61–78)	67.6 (62–77)	7/25	5/20	NA	PLIF	64 (42–90)	
Haddad et al., 2013	Retro	UK, London and Stanmore	2004–2006	40.6 ± 6.46	46.5 ± 10.7	19/13	15/17	NA	PLIF	48	
Yu et al., 2012	RCT	China, Taiwan	2006–2007	52.22 ± 8.31	55.52 ± 6.98	10/17	11/18	1	PLIF	36	

*RCT* randomized controlled trial, *Pro* prospective clinical trial, *Retro* retrospective clinical trial, *NOS* Newcastle–Ottawa Scale, *TLIF* transforaminal lumbar interbody fusion, *PLIF* posterior lumbar interbody fusion, *PLF* posterolateral fusion, *NA* not applicable

### Risk of bias in the included studies

The risk of bias for the included studies are shown in Additional file 2 and Additional file 3. The only randomized controlled trial [21] was of moderate quality and showed adequate randomization and allocation concealment. However, the blinding of participants and personnel and blinding of outcome assessment were of high risk. For the comparative cohort studies assessed by NOS, seven studies [3, 7, 15, 17, 24, 25, 31] were of sufficiently high quality, and nine studies [4, 16, 22, 26, 30, 32–35] were of moderate quality.

## Meta-analysis results

### Clinical outcomes at final follow-up

#### Postoperative VAS scores

**Fig. 2:**
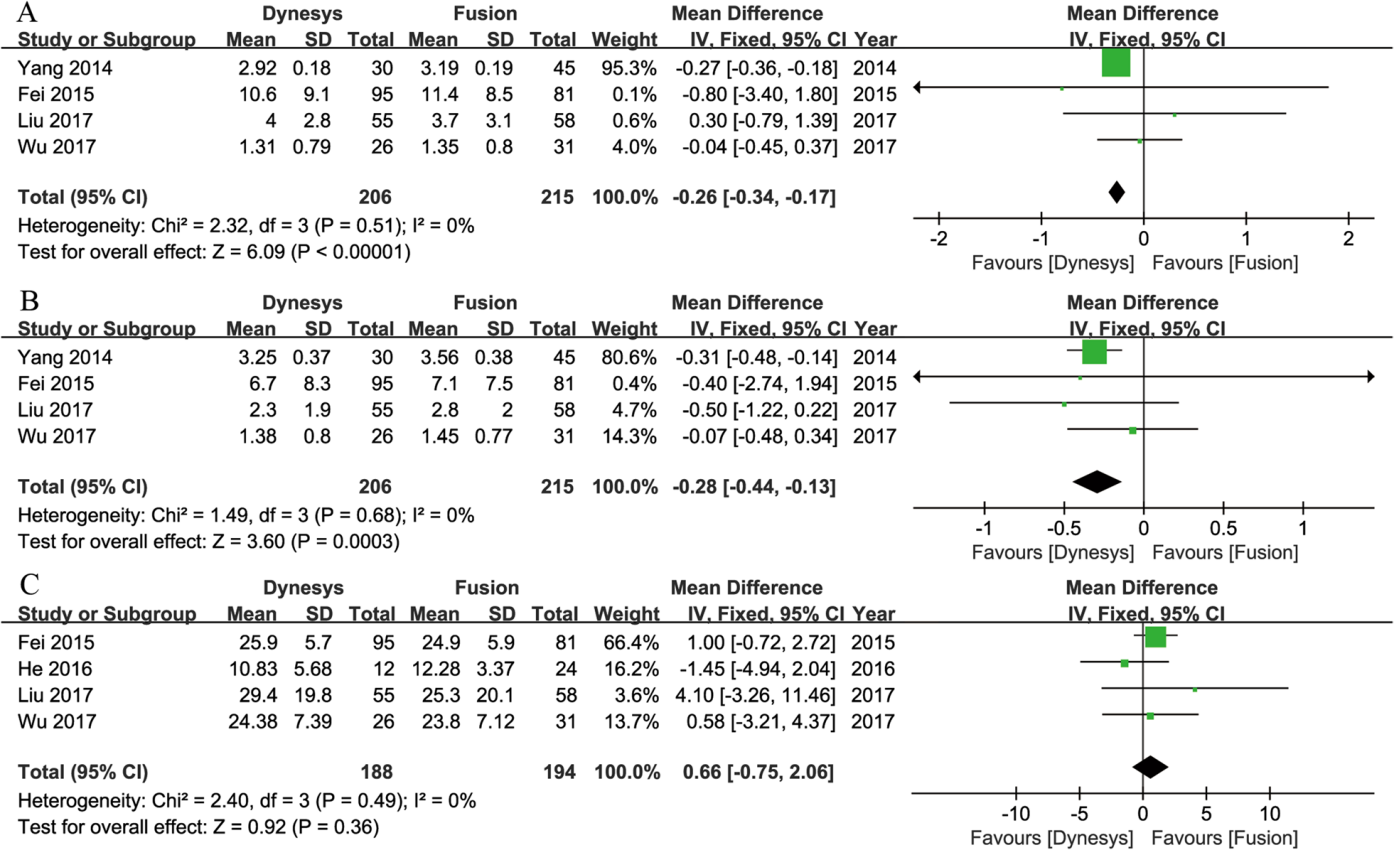
Forest plots of Dynesys stabilization versus instrumented fusion: (**A**) postoperative back VAS scores; (**B**) postoperative leg VAS scores; (**C**) postoperative ODI scores

Four prospective studies [3, 15, 17, 30] provided data on post-operative back pain and leg pain scores between Dynesys and fusion groups. The combined results indicated that the postoperative VAS scores for low back pain (MD = − 0.26, 95% CI of − 0.34 to − 0.17, *P* < 0.001, I^2^ = 0%; Fig. 2A) and leg pain (MD = − 0.28, 95% CI of − 0.44 to − 0.13, *P* < 0.001, I^2^ = 0%; Fig. 2B) in the Dynesys group were better than those in the fusion group.

#### Postoperative ODI scores

Four prospective studies [3, 15, 16, 30] provided data on post-operative ODI scores. The combined results revealed that the Dynesys group was insignificantly different from the fusion group in terms of postoperative ODI scores (MD = 0.66, 95% CI of − 0.75 to 2.06, *P* = 0.36, I^2^ = 0%; Fig. 2C).

#### Screw loosening and breakage

**Fig. 3:**
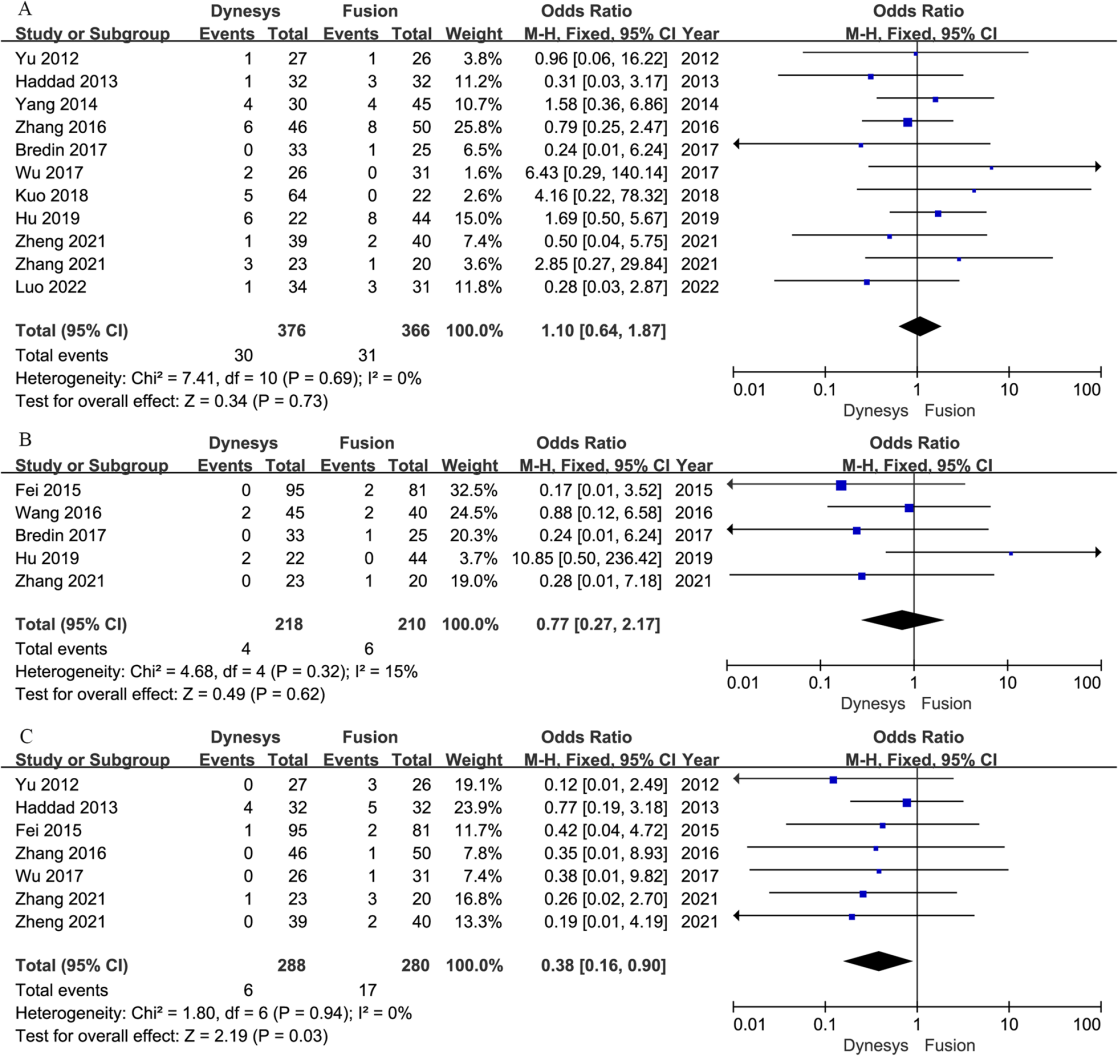
Forest plots of Dynesys stabilization versus instrumented fusion: (**A**) screw loosening; (**B**) screw breakage; (**C**) surgical revision

Eleven studies [7, 15, 17, 21, 22, 24, 26, 31–33, 35] and five studies [3, 4, 7, 33, 35] provided data on screw loosening and breakage, respectively. The pooled results showed that the Dynesys group was insignificantly different from the fusion group in terms of screw loosening (OR = 1.10, 95% CI 0.64–1.87, *P* = 0.73; I^2^ = 0%; Fig. 3A) and screw breakage (OR = 0.77, 95% CI 0.27–2.17, *P* = 0.62; I^2^ = 15%; Fig. 3B). The Egger’s test suggested no publication bias of screw loosening (coefficient = − 0.20, SE = 0.62, *P* = 0.756).

#### Surgical revision

Seven studies [3, 15, 21, 24, 26, 32, 33] provided surgical revision data. The pooled results indicated that the Dynesys group was associated with significantly lower rate of surgical revision than the fusion group (OR = 0.38, 95% CI 0.16–0.90, *P* = 0.03; I^2^ = 0%; Fig. 3C).

### ASP (adjacent segment pathology)

#### ASDeg (radiographic ASD)

**Fig. 4:**
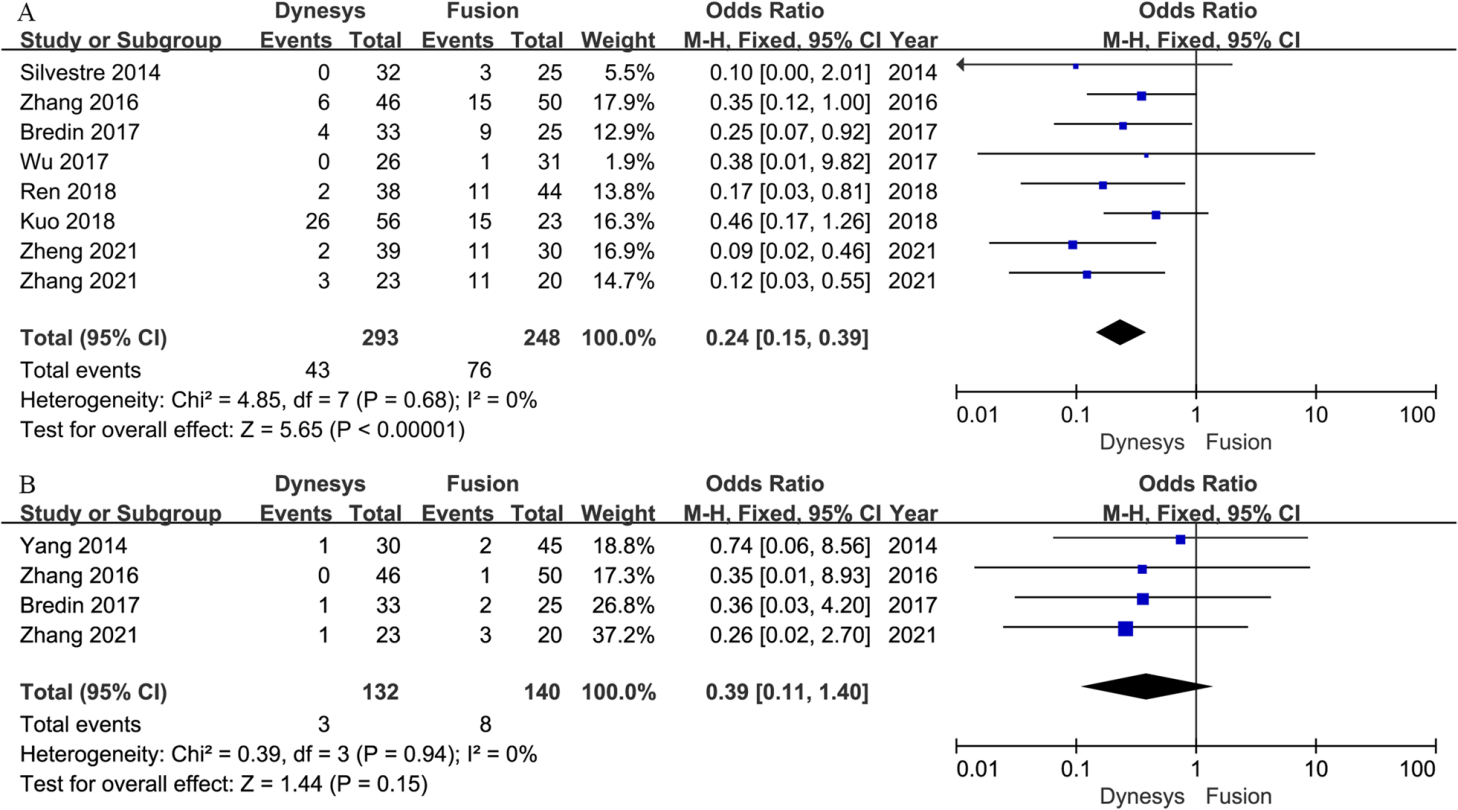
Forest plots of Dynesys stabilization versus instrumented fusion: (**A**) ASDeg; (**B**) ASDis

Eight studies [15, 22, 24, 25, 32–35] provided ASDeg data. The pooled results indicated that the Dynesys group showed less ASDeg than the fusion group (OR = 0.24, 95% CI 0.15–0.39, *P* < 0.001; I^2^ = 0%; Fig. 4A).

#### ASDis (symptomatic ASD)

Four studies [17, 24, 33, 35] provided ASDis data. The pooled results showed that the Dynesys group was insignificantly different from the fusion group in terms of ASDis (OR = 0.39, 95% CI 0.11–1.40, *P* = 0.15; I^2^ = 0%; Fig. 4B).

### Radiographic outcomes

#### Postoperative ROM

**Fig. 5:**
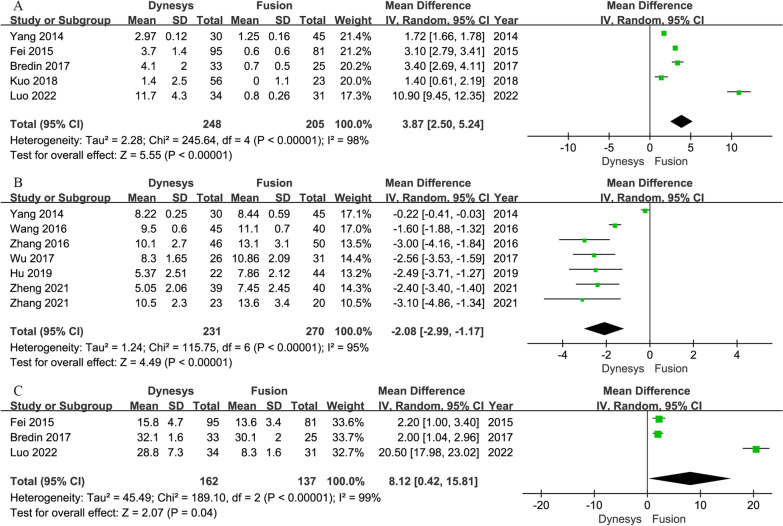
Forest plots of Dynesys stabilization versus instrumented fusion: (**A**) ROM at stabilized segments; (**B**) ROM at proximal adjacent segment; (**C**) ROM of L1–S1 lumbar lordosis angle

Five studies [3, 17, 22, 31, 35] provided data on post-operative ROM at stabilized segments. The pooled data indicated that the ROM at the stabilized segment in the fusion group decreased significantly than that in the Dynesys group (MD = 3.87, 95% CI of 2.50 to 5.24, *P* < 0.001, I^2^ = 98%; Fig. 5A). Sensitivity analysis was performed by sequential removal of the included trials due to the remarkable heterogeneity (I^2^ = 98%). The results showed the same conclusion (MD = 3.15, 95% CI of 2.86 to 3.43, *P* < 0.001, I^2^ = 0%) as before.

Seven studies [4, 7, 15, 17, 24, 32, 33] provided data on post-operative ROM at proximal adjacent segment. The combined results indicated that the ROM in the fusion group increased significantly than that in the Dynesys group (MD = − 2.08, 95% CI of − 2.99 to − 1.17, *P* < 0.001, I^2^ = 95%; Fig. 5B). Sensitivity analysis was performed because of remarkable heterogeneity (I^2^ = 95%). The obtained result (MD = − 2.64, 95% CI of − 3.15 to − 2.12, *P* < 0.001, I^2^ = 0%) was consistent with the above outcomes after removing the studies of Yang et al. [17] and Wang et al. [4] that caused heterogeneity.

Three studies [3, 31, 35] provided data on the post-operative ROM of L1–S1 lumbar lordosis (LL) angle. The combined results indicated that the ROM in the fusion group decreased significantly than that in the Dynesys group (MD = 8.12, 95% CI of 0.42–15.81, *P* = 0.040, I^2^ = 99%; Fig. 5C). After the sensitivity analysis, the final result (MD = 2.08, 95% CI of 1.33–2.83, *P* < 0.001, I^2^ = 0%) confirmed the above outcome.

#### Postoperative disc height

Five studies [7, 15, 17, 24, 33] reported the disc height at the surgical segment, and four studies [7, 15, 24, 33] reported the disc height at the proximal adjacent segment. The pooled results showed that the Dynesys group was insignificantly different from the fusion group in terms of the disc height at the surgical segment (MD = − 0.53, 95% CI of − 1.30 to 0.23, *P* = 0.170, I^2^ = 83%; Additional file 4: Fig. S1A) and the disc height at the proximal adjacent segment (MD = − 0.23, 95% CI of − 0.66 to 0.20, *P* = 0.300, I^2^ = 0%; Additional file 4: Fig. S1B).

## Discussion

Spine fusion is a primary therapy for spinal degenerative diseases, but this method is associated with several complications, especially the acceleration of ASP [1, 5, 13]. In response to the complications caused by fusion, dynamic stabilization techniques have been developed, including DS, which is a widely used non-fusion technique with the advantage of ASP prevention [3].

Many studies have confirmed the safety and clinical equivalence of DS to fusion method. [4, 21, 24, 36] DS relieves clinical symptoms and improves the functional status and fusion instruments with durations of follow-up ranging from 28.78 months to 70.14 months [7, 15, 16, 20, 26]. Furthermore, Bredin et al. [35] performed a study with a follow-up of 93.6 months and concluded that the DS group showed substantial improvements in VAS and ODI scores compared with the fusion method. The fusion method is likely to be chosen in the retrospective cases, in which a more severe disease state is present. Additionally, patients with far lateral discs or other pathology that required facetectomy and TLIF were not considered as candidates for DS because of the possibility of introducing confounding variables. Therefore, only the prospective studies that report the VAS and ODI scores were included for analyses, because these studies may effectively control the severity of diseases in this meta-analysis. In conclusion, DS remarkably improved the VAS scores for back and leg pain. No significant difference was observed in post-operative ODI scores between the two groups. The results may be explained as follows. Although the decompression of nerve roots was conducted in DS and fusion methods, the risk of nerve root injuries increased, because the latter not only dissects the bone and soft tissue but also requires the preparation of endplates and insertion of interbody devices or bone grafting, leading to back and leg pain after surgery. Additional surgical instrument and enlargement of surgical trauma in fusion method may increase the surgical time, blood loss, and in-hospital complications. Furthermore, the occurrence of non-fusion with fusion method would aggravate clinical symptoms in the later recovery process. Moreover, the Dynesys group did not differ from the fusion group in terms of screw loosening and breakage. These results can be attributed to the simplified operation of DS and the preservation of lumbar mobility. Hence, DS showed equivalent, or even better clinical measurements, compared with conventional fusion. The safety of DS has complied with the basic requirements for widespread clinical application in cases meeting indications, such as disc herniation, lumbar spinal stenosis, and grade I degenerative spondylolisthesis in lumbar.

DS is mainly used to minimize ASP. [6, 37] Hashimoto et al. [8] reported that the fusion of stabilized segments may increase the biomechanical stress on the adjacent levels, leading to ASDeg and ASDis. DS preserves the ROM at the stabilized segments and prevents hypermobility at the adjacent segments in the medium or long-term follow-up [4, 7, 15, 20, 21, 24, 36]. By contrast, Yang et al. [17] found that DS does not maintain the ROM at the adjacent segments compared with fusion method after a minimum follow-up of 24 months. Furthermore, Schaeren et al. [38] found no measurable motion at the stabilized segments after DS but reported signs of degeneration at adjacent segments in 47% patients after 4 years, thus supporting our findings from the use of fusion instrument. In the current meta-analysis, in the fusion group, the ROM at stabilized segments and the ROM of LL decreased remarkably, but the ROM at adjacent segments increased remarkably compared with those in the DS group. This result was obtained, possible because the DS pedicle screws are connected to the PET cords, thereby providing tension to limit excessive flexion and PCU spacers resisting compressive force to limit over extension; this phenomenon leads to reduced vertebral abnormal activity and preserved motion at the instrumental segments and hypermobility at the adjacent segments in the medium and long-term duration [39].

Another early radiographic manifestation of ASP in clinical practice is the narrowness and loss of the intervertebral space, and its height is commonly considered as an indicator to evaluate the degree of ASP [3, 15]. In the current meta-analysis, the Dynesys and fusion group did not differ in terms of disc heigh both at stabilized and adjacent segments. The results can be attributed to the natural degenerative progression of the disc at the stabilized and adjacent segments despite DS, and this phenomenon is not associated with the stabilization method [40, 41]. Thus, DS showed no advantage over fusion method in terms of prevention of disc degeneration after surgery.

Whether DS can delay the occurrence of ASP has not been confirmed. In the study of Bredin et al. [35], a mean follow-up of 5.5 years was obtained, and significantly less ASDeg was observed in the DS group than in the fusion group (12.1% versus 36%); Zhang et al. [24] confirmed the conclusion in their study with mean follow-up of 55.2 months. DS may not prevent ASDeg, with high rates ranging from 16 to 47% [37, 38, 42]. In the current meta-analysis, the Dynesys group showed a lower rate of ASDeg than the fusion group. Although DS showed no superiority in terms of preventing disc degeneration compared with fusion method, the former performed well in terms of sparing abnormal biomechanical load at the stabilized and adjacent segments, consequently preserving the physiological motion after stabilization; this finding may explain why DS can prevent ASDeg [10, 43]. Furthermore, the PET cords and PCU spacers were used to restrict flexion and extension. With increasing cord pretension, the flexion ROM at stabilized and adjacent levels increased, but the extension ROM decreased [44]. The realistic stiffness of DS varies with cord pretension and spacer length, which are decided based on surgeons’ personal experience; this phenomenon might partly explain the conflicting results in previously published works [7, 45, 46].

The prevention of ASDis is crucial because of the potential risk of revision surgery and adverse effect on health care outcomes and cost. Our meta-analysis demonstrated that the Dynesys group showed less ASDis than the fusion group, whereas the difference was not significant. The result revealed that DS can prevent ASDeg instead of ASDis compared with the fusion method. The occurrence of ASDis is relatively lower than that of ASDeg, because ASDeg does not always cause clinical symptoms. Furthermore, the lack of remarkable difference in disabilities (ODI) post-operatively between the two methods indicate the similar incidence of ASDis after surgery.

Screw loosening is a common complication of DS. The current study also showed that the Dynesys group did not differ from the fusion group in terms of screw loosening and screw breakage. Ko et al. [47] included 71 patients and found that the screw loosening had no adverse effect on the improvement of VAS and ODI scores after the surgery. Hu et al. [48] investigated the mid- and long-term outcomes of hybrid surgery that combined Dynesys fusion and non-fusion stabilization in the treatment of degenerative lumbar diseases. The results show that screw loosening mainly occurred at the end of the fixed segment, and old age was a risk factor for screw loosening. Screw loosening is usually asymptomatic and can be observed regularly. If the symptoms related to screw loosening or screw breakage occur, and the conservative treatment fails, revision surgery is required. Based on Chinese expert consensus on the treatment of lumbar degenerative disease by trans-pedicle dynamic rod fixation, the rate of screw loosening can be reduced by preserving the integrity of the bony structure and ligaments of the posterior column during decompression; thicker and longer screws are preferred, and repeated adjustment of depth and direction need to be avoided when placing screws [49].

The disadvantages of DS should be highlighted. DS is designed to preserve lumbar vertebral mobility and reduce the load on the intervertebral disc; if these two points cannot be achieved, the use of this technique has no advantage [49]. The Dynesys device is subjected to continuous stress during weight-bearing of the spine. Thus, fixation failure may occur in the cases of severe osteoporosis and severe decreases of lumbar stabilization. Besides, DS cannot effectively stabilize the lumbar spine when used for isthmic spondylolisthesis with a high risk of fixation failure [50]. Moreover, for patients with severe stenosis in intervertebral space and small range of motion before surgery, the range of lumbar motion is limited after surgery, thus rendering the use of DS unsuitable [51].

This meta-analysis has several limitations. First, only one RCT along with 16 comparative cohort studies was included, resulting in less powerful results compared with that obtained purely from RCTs. Second, the definitions of ASDed and ASDis in previous studies are inconsistent and ambiguous. No consensus criterion has been established to define ASDed and ASDis, leading to imprecise outcome measures. Third, the number of the included studies was limited in terms of the ROM of LL, disc heigh at proximal adjacent segments, and ASDis. Furthermore, the fusion method is likely to encounter screw loosening initially, while the dynamic stabilization is likely to fail in the latter part. This flaw is expected in studies that evaluate early outcomes. Thus, future work should employ more RCTs of high quality and patients with follow-up more than 10 years from different cultural contexts.

## Conclusion

DS showed equivalent outcomes in terms of ODI, screw loosing, screw breakage, and ASDis compared with fusion method. Moreover, DS had better clinical measurements in terms of VAS scores for back and leg pain than fusion instrument. DS showed comparable clinical outcomes and provided benefits in preserving the motion at the stabilized segments, thus limiting the hypermobility at the adjacent segments and preventing ASDeg compared with the fusion method in degenerative disease with or without grade I spondylolisthesis.

## Supplementary Information

**Additional file 1.** **Table S1.** Search strategy for each database.

**Additional file 2.** **Table S2.** Risk of bias assessment of the RCT study.

**Additional file 3.** **Table S3.** Risk of bias assessment of the cohort studies.

**Additional file 4.** **Fig. S1.** Forest plots of Dynesys stabilization versus instrumented fusion: (A) disc height at the intervertebral segment; (B) disc height at the proximal adjacent segment.

## Data Availability

All data generated or analyzed during this study are included in this published article and its Additional information files.

## Abbreviations

DS: Dynesys stabilization
ROM: Range of motion
ASDeg: Adjacent segment degeneration
ASDis: Adjacent segment disease
ASP: Adjacent segment pathology
PET: Polyethylene–terephtalate
PCU: Polycarbonate–urethane
PRISMA: The Preferred Reporting Items for Systematic Reviews and Meta-analyses
VAS: Visual analogue scale
ODI: Oswestry disability index
RCTs: Randomized controlled trials
NOS: Newcastle–Ottawa Scale
RR: Risk ratio
MD: Mean difference
CI: Confidence interval

## References

[1] Yavin D, Casha S, Wiebe S, Feasby TE, Clark C, Isaacs A, Holroyd-Leduc J, Hurlbert RJ, Quan H, Nataraj A (2017). Lumbar fusion for degenerative disease: a systematic review and meta-analysis. Neurosurgery.

[2] Lee JC, Choi S-W (2015). Adjacent segment pathology after lumbar spinal fusion. Asian Spine J.

[3] Fei H, Xu J, Wang S, Xie Y, Ji F, Xu Y (2015). Comparison between posterior dynamic stabilization and posterior lumbar interbody fusion in the treatment of degenerative disc disease: a prospective cohort study. J Orthop Surg Res.

[4] Wang Q, Liu J, Shi Y, Chen Y, Yu H, Ma J, Ren W, Yang H, Wang H, Xiang L (2016). Short-term effects of a dynamic neutralization system (Dynesys) for multi-segmental lumbar disc herniation. Eur Spine J.

[5] Zhang Y, Zhang ZC, Li F, Sun TS, Shan JL, Guan K, Zhao GM, Zhang LZ (2018). Long-term outcome of dynesys dynamic stabilization for lumbar spinal stenosis. Chin Med J (Engl).

[6] Zhang C, Berven SH, Fortin M, Weber MH (2016). Adjacent segment degeneration versus disease after lumbar spine fusion for degenerative pathology. Clin Spine Surg.

[7] Hu A, Sun C, Liang Y, Wang H, Li X, Dong J (2019). Multi-segmental lumbar spinal stenosis treated with Dynesys stabilization versus lumbar fusion in elderly patients: a retrospective study with a minimum of 5 years' follow-up. Arch Orthop Trauma Surg.

[8] Hashimoto K, Aizawa T, Kanno H, Itoi E (2019). Adjacent segment degeneration after fusion spinal surgery-a systematic review. Int Orthop.

[9] Huang Y-J, Zhao S-J, Zhang Q, Nong L-M, Xu N-W (2017). Comparison of lumbar pedicular dynamic stabilisation systems versus fusion for the treatment of lumbar degenerative disc disease: a meta-analysis. Acta Orthop Belg.

[10] Lee SE, Jahng T-A, Kim H-J (2016). Clinical experiences of non-fusion dynamic stabilization surgery for adjacent segmental pathology after lumbar fusion. Int J Spine Surg.

[11] Donnally CJ, Patel PD, Canseco JA, Divi SN, Goz V, Sherman MB, Shenoy K, Markowitz M, Rihn JA, Vaccaro AR (2020). Current incidence of adjacent segment pathology following lumbar fusion versus motion-preserving procedures: a systematic review and meta-analysis of recent projections. Spine J.

[12] Fayyazi AH, Ordway NR, Park S-A, Fredrickson BE, Yonemura K, Yuan HA (2010). Radiostereometric analysis of postoperative motion after application of Dynesys dynamic posterior stabilization system for treatment of degenerative spondylolisthesis. J Spinal Disord Tech.

[13] Akyoldas G, Cevik OM, Suzer T, Sasani M, Oktenoglu T, Ozer AF (2020). Dynamic stabilization of the lumbar spine using the dynesys system. Turk Neurosurg.

[14] Jung J-M, Hyun S-J, Kim K-J, Jahng T-A (2021). Dynamic stabilization surgery in patients with spinal stenosis: Long-term outcomes and the future. Spine.

[15] Wu H, Pang Q, Jiang G (2017). Medium-term effects of Dynesys dynamic stabilization versus posterior lumbar interbody fusion for treatment of multisegmental lumbar degenerative disease. J Int Med Res.

[16] He J, Li J, Luo C, Sun Y, Nong L, Xie H (2016). Impact of the Dynesys dynamic stabilization system on the fixation-adjacent intervertebral discs. Int J Clin Exp Med.

[17] Yang M, Li C, Chen Z, Bai Y, Li M (2014). Short term outcome of posterior dynamic stabilization system in degenerative lumbar diseases. Indian J Orthop.

[18] Zhao C, Liu L, Luo L, Li P, Wang Y, Liang L, Wen X, Jiang D, Zhou Q (2021). Effect of discectomy on Dynesys dynamic fixation in the treatment of lumbar degenerative diseases. Pain Res Manag.

[19] Luo L, Zhao C, Li P, Liu L, Zhou Q, Luo F, Liang L (2021). Posterior dynamic stabilization with limited rediscectomy for recurrent lumbar disc herniation. Pain Res Manag.

[20] Zhang C, Wang L, Hou T, Luo L, Zhao C, Gan Y, Zhou Q, Li P (2017). The influence of L4–S1 Dynesys® dynamic stabilization versus fusion on lumbar motion and its relationship with lumbar degeneration: a retrospective study. J Orthop Surg Res.

[21] Yu S-W, Yang S-C, Ma C-H, Wu C-H, Yen C-Y, Tu Y-K (2012). Comparison of Dynesys posterior stabilization and posterior lumbar interbody fusion for spinal stenosis L4L5. Acta Orthop Belg.

[22] Kuo C-H, Huang W-C, Wu J-C, Tu T-H, Fay L-Y, Wu C-L, Cheng H (2018). Radiological adjacent-segment degeneration in L4–5 spondylolisthesis: comparison between dynamic stabilization and minimally invasive transforaminal lumbar interbody fusion. J Neurosurg Spine.

[23] St-Pierre GH, Jack A, Siddiqui MMA, Henderson RL, Nataraj A (2016). Nonfusion does not prevent adjacent segment disease: Dynesys long-term outcomes with minimum five-year follow-up. Spine.

[24] Zhang Y, Shan J-L, Liu X-M, Li F, Guan K, Sun T-S (2016). Comparison of the dynesys dynamic stabilization system and posterior lumbar interbody fusion for lumbar degenerative disease. PLoS ONE.

[25] Silvestre MD, Lolli F, Bakaloudis G (2014). Degenerative lumbar scoliosis in elderly patients: dynamic stabilization without fusion versus posterior instrumented fusion. Spine J.

[26] Haddad B, Makki D, Konan S, Park D, Khan W, Okafor B (2013). Dynesys dynamic stabilization: less good outcome than lumbar fusion at 4-year follow-up. Acta Orthop Belg.

[27] Liberati A, Altman DG, Tetzlaff J, Mulrow C, Gøtzsche PC, Ioannidis JPA, Clarke M, Devereaux PJ, Kleijnen J, Moher D (2009). The PRISMA statement for reporting systematic reviews and meta-analyses of studies that evaluate health care interventions: explanation and elaboration. PLoS Med.

[28] Higgins JPT, Green S. Cochrane handbook for systematic reviews of interventions version 5.1.0. The Cochrane Collaboration, 2011. http://www.cochrane-handbook.org. Accessed March 20, 2012.

[29] Wells GA, Shea B, O’Connell D, Robertson J, Peterson J, Welch V, Losos M, Tugwell P. The Newcastle-Ottawa Scale (NOS) for assessing the quality of nonrandomized studies in meta-analysis. 2011. http://www.ohri.ca/programs/clinicalepidemiology/oxford.asp Accessed April 15, 2012.

[30] Liu K, Sun W, Lu Q, Chen J, Tang J (2017). A cost-utility analysis of Dynesys dynamic stabilization versus instrumented fusion for the treatment of degenerative lumbar spine diseases. J Orthop Sci.

[31] Luo L, Liu L, Li P, Zhao C, Liang L, Luo F, Zhou Q, Chen Y, Fang L (2022). Comparison between dynamic stabilization and instrumented fusion in the treatment of spinal stenosis with degenerative lumbar scoliosis. Pain Res Manag.

[32] Zheng C, Liu J, Du J, Ma W, Chen Y, Wu J (2021). Application of Dynesys dynamic stabilization with microendoscopic discectomy for the degenerative lumbar spinal stenosis in the elder. Chin J Orthop.

[33] Zhang K, Luo K, Cai K, Lu B, Lu J, Jiang G, Wu H (2021). Medium and long⁃term comparisons of Dynesys stabilization and posterior lumbar interbody fusion for two⁃level lum⁃ bar degenerative diseases. Chin J Orthop.

[34] Ren D-W, Li Q, Jia T, He Y (2018). Comparison of the Dynesys dynamicinternal system and transforminal lumbar interbody fusion for degenerative L5/S1 disc herniation. Orthopaedics.

[35] Bredin S, Demay O, Mensa C, Madi K, Ohl X (2017). Posterolateral fusion versus Dynesys dynamic stabilization: retrospective study at a minimum 5.5 years’ follow-up. Orthop Traumatol Surg Res.

[36] Veresciagina K, Mehrkens A, Schären S, Jeanneret B (2018). Minimum ten-year follow-up of spinal stenosis with degenerative spondylolisthesis treated with decompression and dynamic stabilization. J Spine Surg.

[37] Hoppe S, Schwarzenbach O, Aghayev E, Bonel H, Berlemann U (2012). Long-term outcome after monosegmental L4/5 stabilization for degenerative spondylolisthesis with the Dynesys device. Clin Spine Surg.

[38] Schaeren S, Broger I, Jeanneret B (2008). Minimum four-year follow-up of spinal stenosis with degenerative spondylolisthesis treated with decompression and dynamic stabilization. Spine.

[39] Más Y, Gracia L, Ibarz E, Gabarre S, Peña D, Herrera A (2017). Finite element simulation and clinical follow-up of lumbar spine biomechanics with dynamic fixations. PLoS ONE.

[40] Kumar A, Beastall J, Hughes J, Karadimas EJ, Nicol M, Smith F, Wardlaw D (2008). Disc changes in the bridged and adjacent segments after Dynesys dynamic stabilization system after two years. Spine.

[41] Klöckner C (2010). Long-term results of the Dynesys implant. Orthopade.

[42] Hoff E, Strube P, Gross C, Putzier M (2013). Sequestrectomy with additional transpedicular dynamic stabilization for the treatment of lumbar disc herniation: no clinical benefit after 10 years follow-up. Spine.

[43] Shih S-L, Liu C-L, Huang L-Y, Huang C-H, Chen C-S (2013). Effects of cord pretension and stiffness of the dynesys system spacer on the biomechanics of spinal decompression- a finite element study. BMC Musculoskelet Disord.

[44] Mesbah M, Barkaoui A (2020). Biomechanical investigation of the effect of pedicle-based hybrid stabilization constructs: a finite element study. Proc Inst Mech Eng H.

[45] Lee C-H, Jahng T-A, Hyun S-J, Kim CH, Park S-B, Kim K-J, Chung CK, Kim H-J, Lee S-E (2016). Dynamic stabilization using the Dynesys system versus posterior lumbar interbody fusion for the treatment of degenerative lumbar spinal disease: a clinical and radiological outcomes-based meta-analysis. Neurosurg Focus.

[46] Jahng T-A, Kim YE, Moon KY (2013). Comparison of the biomechanical effect of pedicle-based dynamic stabilization: a study using finite element analysis. Spine J.

[47] Ko CC, Tsai HW, Huang WC, Wu JC, Chen YC, Shih YH, Chen HC, Wu CL, Cheng H (2010). Screw loosening in the Dynesys stabilization system: radiographic evidence and effect on outcomes. Neurosurg Focus.

[48] Hu A, Chen F, Jiang L, Jiang Y, Lin H, Li X, Zhou X, Dong J (2021). Mid- and long-term outcomes of hybrid surgery combined Dynesys fusion and non-fusion stabilization in the treatment of degenerative lumbar diseases. Chin J Orthop.

[49] The Lumbar Research Group of Spinal Cord Committee of Chinese Association of Rehabilitation Medicine. Expert consensus on the treatment of lumbar degenerative disease by transpedicle dynamic rod fixation. Chin J Orthop 2020; 40(24):1639–1645.

[50] Guo J, Hou S, Shi Y, Li L, Wang H (2013). Early clinical effects of Dynesys dynamic internal fixation system for mild lumbar degenerative spondylolisthesis. Chin J Bone Joint.

[51] Schwaiger BJ, Behr M, Gersing AS, Meyer B, Zimmer C, Kirschke JS, Ryang YM, Ringel F (2016). Computed tomography findings associated with clinical outcome after dynamic posterior stabilization of the lumbar spine. World Neurosurg.

