# Supplementary material for: Medium and long-term radiographic and clinical outcomes of Dynesys dynamic stabilization versus instrumented fusion for degenerative lumbar spine diseases
Source: BMC Surg. 2023 Feb 28;23:46. doi: 10.1186/s12893-023-01943-6 (PMC9976523; doi:10.1186/s12893-023-01943-6)
Supplement: Supplementary file 1 — Additional file 1. Table S1. Search strategy for each database. [file 12893_2023_1943_MOESM1_ESM.docx]

**Table S1. Search strategy for each database**

| **Database** | **Search strategy** |
| --- | --- |
| Pubmed | #1 "dynamic"[All Fields] OR "semi-rigid"[All Fields] OR "Dynesys"[All Fields]  #2 "fusion"[All Fields]  #3 "lumbar"[All Fields]  #4 #1 AND #2 AND #3 |
| Embase | #1 "dynamic"[All Fields] OR "semi-rigid"[All Fields] OR "Dynesys"[All Fields]  #2 "fusion"[All Fields]  #3 "lumbar"[All Fields]  #4 #1 AND #2 AND #3 |
| Cochrane library | #1 "dynamic"[TI,AB,KW] OR "semi-rigid"[TI,AB,KW] OR "Dynesys"[TI,AB,KW]  #2 "fusion"[TI,AB,KW]  #3 "lumbar"[TI,AB,KW]  #4 #1 AND #2 AND #3 |
| Web of Science | #1 "dynamic"[TS] OR "semi-rigid"[TS] OR "Dynesys"[TS]  #2 "fusion"[TS]  #3 "lumbar"[TS]  #4 #1 AND #2 AND #3 |
| Chinese National Knowledge Databases | #1 “Dynesys” [TS]  #2 “ji zhu” [TS]  #3 #1 AND #2 |
| Wanfang Database | #1 “Dynesys” [TS]  #2 “ji zhu” [TS]  #3 #1 AND #2 |
