# Supplementary material for: Medium and long-term radiographic and clinical outcomes of Dynesys dynamic stabilization versus instrumented fusion for degenerative lumbar spine diseases
Source: BMC Surg. 2023 Feb 28;23:46. doi: 10.1186/s12893-023-01943-6 (PMC9976523; doi:10.1186/s12893-023-01943-6)
Supplement: Supplementary file 2 — Additional file 2. Table S2. Risk of bias assessment of the RCT study. [file 12893_2023_1943_MOESM2_ESM.docx]

**Table S2. Risk of bias assessment of the RCT study**

| **RCT** | **Random sequence generation** | **Allocation**  **concealment** | **Blinding of participants and personnel** | **Blinding of outcome assessment** | **Incomplete outcome data** | **Selective reporting** | **Other bias** |
| --- | --- | --- | --- | --- | --- | --- | --- |
| Yu et al., 2012 | Low risk | Low risk | High risk | Unclear risk | Low risk | Unclear risk | Low risk |

Other bias: the baseline characteristics in the experimental and control groups were different.
