# Supplementary material for: Medium and long-term radiographic and clinical outcomes of Dynesys dynamic stabilization versus instrumented fusion for degenerative lumbar spine diseases
Source: BMC Surg. 2023 Feb 28;23:46. doi: 10.1186/s12893-023-01943-6 (PMC9976523; doi:10.1186/s12893-023-01943-6)
Supplement: Supplementary file 3 — Additional file 3. Table S3. Risk of bias assessment of the cohort studies. [file 12893_2023_1943_MOESM3_ESM.docx]

**Table S3. Risk of bias assessment of the cohort studies**

| Cohort | Selection | Comparability | Outcome | Total |
| --- | --- | --- | --- | --- |
| Luo et al., 2022 | ★★★ | ★★ | ★★★ | ★★★★★★★★ |
| Zheng et al., 2021 | ★★★ | ★★ | ★★ | ★★★★★★★ |
| Zhang et al., 2021 | ★★★ | ★★ | ★★ | ★★★★★★★ |
| Hu et al., 2019 | ★★★ | ★★ | ★★★ | ★★★★★★★★ |
| Kuo et al., 2018 | ★★★ | ★★ | ★ | ★★★★★★ |
| Ren et al., 2018 | ★★★ | ★ | ★★ | ★★★★★★ |
| Wu et al., 2017 | ★★★★ | ★ | ★★★ | ★★★★★★★★ |
| Liu et al., 2017 | ★★★★ | ★★ | ★ | ★★★★★★★ |
| Bredin et al., 2017 | ★★★ | ★★ | ★★ | ★★★★★★★ |
| Zhang et al., 2016 | ★★★ | ★★ | ★★★ | ★★★★★★★★ |
| He et al., 2016 | ★★★★ | ★ | ★ | ★★★★★★ |
| Wang et al., 2016 | ★★★ | ★ | ★★ | ★★★★★★ |
| Fei et al., 2015 | ★★★★ | ★★ | ★★ | ★★★★★★★★ |
| Yang et al., 2014 | ★★★★ | ★★ | ★★ | ★★★★★★★★ |
| Silvestre et al., 2014 | ★★★ | ★★ | ★★★ | ★★★★★★★★ |
| Haddad et al., 2013 | ★★★ | ★★ | ★★ | ★★★★★★★ |
