# Supplementary material for: Medium and long-term radiographic and clinical outcomes of Dynesys dynamic stabilization versus instrumented fusion for degenerative lumbar spine diseases
Source: BMC Surg. 2023 Feb 28;23:46. doi: 10.1186/s12893-023-01943-6 (PMC9976523; doi:10.1186/s12893-023-01943-6)

**Supplementary File 4.** Forest plots of Dynesys stabilization versus instrumented fusion: (A) disc height at the intervertebral segment; (B) disc height at the proximal adjacent segment.


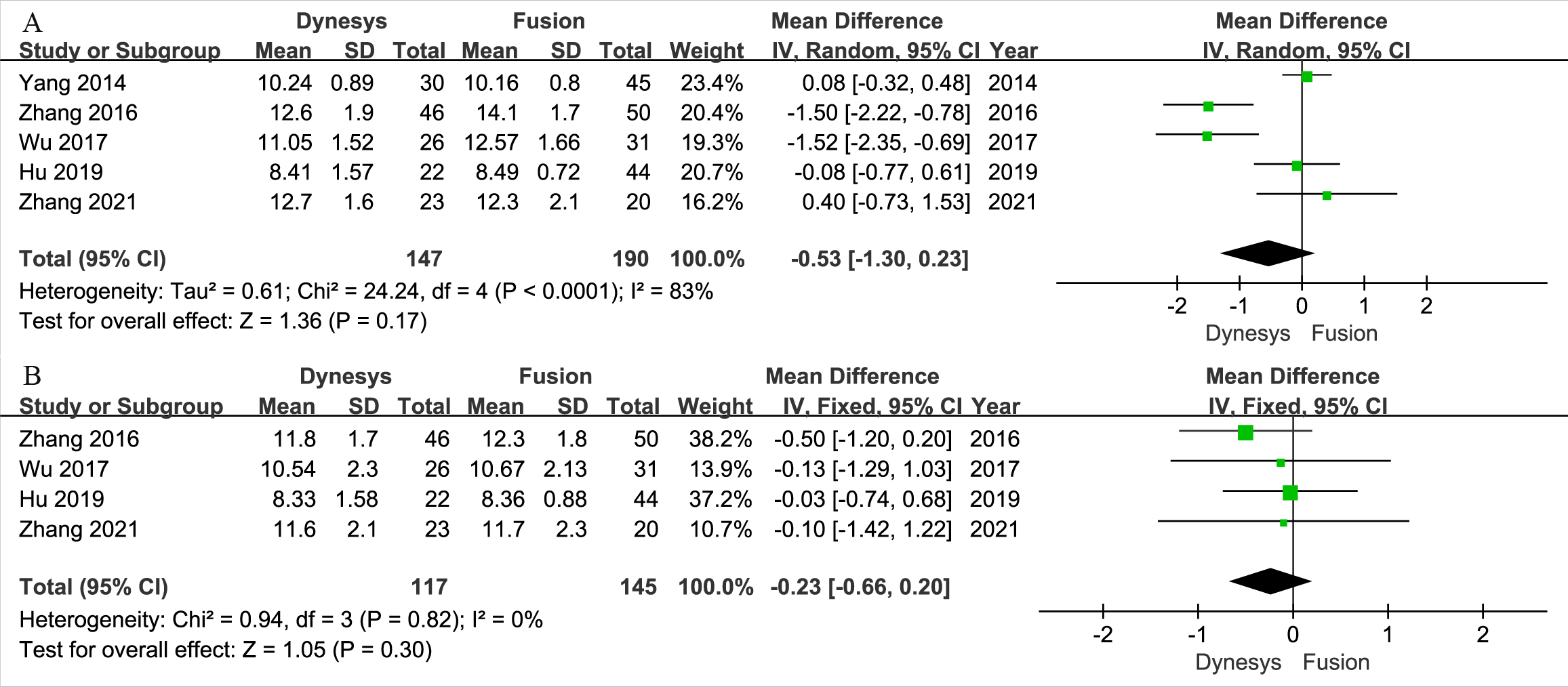

Supplement: Supplementary file 4 — Additional file 4. Fig. S1. Forest plots of Dynesys stabilization versus instrumented fusion: (A) disc height at the intervertebral segment; (B) disc height at the proximal adjacent segment. [file 12893_2023_1943_MOESM4_ESM.docx]
